# Inhibition of LpxC Increases the Activity of Iron Chelators and Gallium Nitrate in Multidrug-Resistant *Acinetobacter baumannii*

**DOI:** 10.3390/antibiotics10050609

**Published:** 2021-05-20

**Authors:** Víctor Vinuesa, Raquel Cruces, Francesca Nonnoi, Michael J. McConnell

**Affiliations:** 1 Intrahospital Infections Laboratory, National Centre for Microbiology, Instituto de Salud Carlos III, 28220 Madrid, Spain; vvinuesa@isciii.es (V.V.); rcrufer@gmail.com (R.C.); francesca.nonnoi@gmail.com (F.N.); 2 Vaxdyn S.L., Avenida Manuel Siurot s/n., 41010 Seville, Spain

**Keywords:** LpxC inhibitors, iron chelators, gallium, antibiotic resistance, synergy

## Abstract

Infections caused by multidrug-resistant *Acinetobacter baumannii* would benefit from the development of novel treatment approaches. Compounds that interfere with bacterial iron metabolism, such as iron chelators and gallium nitrate, have previously been shown to have antimicrobial activity against *A. baumannii*. In this study, we characterize the effect of LpxC inhibitors on the antimicrobial activity of previously characterized iron chelators, 2,2′-bipyridyl (BIP) and deferiprone (DFP), and gallium nitrate (Ga(NO_3_)_3_) against *A. baumannii* reference strains and multidrug-resistant clinical isolates. The LpxC inhibitor LpxC-2 was synergistic with BIP for 30% of strains tested (FICI values: 0.38–1.02), whereas inhibition with LpxC-4 was synergistic with BIP for 60% of strains tested (FICI values: 0.09–0.75). In time–kill assays, combinations of BIP with both LpxC inhibitors demonstrated synergistic activity, with a more than 3 log_10_ reduction in bacterial counts compared to BIP alone. LpxC-2 was synergistic with Ga(NO_3_)_3_ for 50% of strains tested (FICI values: 0.27–1.0), whereas LpxC-4 was synergistic with Ga(NO_3_)_3_ for all strains tested (FICI values: 0.08–≤0.50). In time–kill assays, combinations of Ga(NO_3_)_3_ with LpxC-2 and LpxC-4 decreased the growth of both strains compared to each compound separately; however, only the combination with LpxC-4 met the defined criteria for synergy. These results identify a novel synergy between two antimicrobial classes against *A. baumannii* strains.

## 1. Introduction

The emergence and dissemination of infections caused by multidrug-resistant *Acinetobacter baumannii* warrants the development of new treatment and prevention strategies [1]. The identification of compounds that can increase the activity of existing antimicrobials and the characterization of combinations of compounds that demonstrate synergistic activity against multidrug-resistant *A. baumannii* may represent a promising approach. Inhibitors of LpxC, a zinc-dependent deacetylase that catalyzes one of the initial reactions in the biosynthesis of lipid A, are currently being developed as antimicrobial agents for the treatment of infections caused by Gram-negative pathogens [2]. LpxC inhibitors demonstrate potent activity against multiple species associated with multidrug resistance, including *Pseudomonas aeruginosa*, *Escherichia coli* and *Klebsiella pneumoniae*. However, these compounds are generally less active against *A. baumannii* isolates, in spite of the fact that LpxC is required for lipid A biosynthesis in *A. baumannii* and that the enzymatic activity of *A. baumannii* LpxC is inhibited by LpxC inhibitors, which demonstrate activity against other Gram-negative species [3,4,5]. This difference is likely due to the viability of *A. baumannii* in the absence of lipid A/lipooligosaccharide (LOS) biosynthesis, as indicated by multiple studies demonstrating the growth and survival of *A. baumannii* strains completely deficient in lipid A due to mutations in the *lpxA*, *lpxC* and *lpxD* genes [6,7,8]. This is in contrast to the majority of Gram-negative species, which require lipid A biosynthesis for viability [9].

Iron is required for multiple bacterial processes and is thus essential for establishing host infection. For this reason, iron chelators have been explored as potential antimicrobial agents for multiple bacterial species in both in vitro and in vivo studies [10]. Multiple iron-chelating compounds have demonstrated antimicrobial activity against *A. baumannii*, including 2,2′-bipyridyl (BIP), deferiprone (DFP) and DIBI, among others (reviewed in [10]). In addition, gallium, an iron mimetic that interferes with bacterial iron metabolism, has demonstrated activity against different *A. baumannii* strains [11,12,13], and has been evaluated as an antimicrobial agent in a clinical trial [14].

In a previous study, we showed that *A. baumannii* strains deficient in lipid A biosynthesis due to mutations in *lpxA*, *lpxC* and *lpxD* genes demonstrate decreased growth, compared to wild-type parental strains in the presence of the iron chelator BIP [8]. Based on these findings, we hypothesize that pharmacologic inhibition of lipid A biosynthesis using LpxC inhibitors may increase the susceptibility of *A. baumannii* to iron chelators and gallium-containing compounds. In this study, we characterize the effect of LpxC inhibition on the antimicrobial activity of previously characterized iron chelators, 2,2′-bipyridyl (BIP) and deferiprone (DFP), and gallium nitrate (Ga(NO_3_)_3_ against *A. baumannii* reference strains and multidrug-resistant clinical isolates.

## 2. Results

### 2.1. Checkerboard Assays with A. baumannii Reference Strains

In order to initially characterize potential synergistic activity between LpxC inhibitors and iron-chelating or gallium compounds, the *A. baumannii* reference strains ATCC 179878 and ATCC 19606 were employed in checkerboard assays with the iron chelators BIP, DFP and Ga(NO_3_)_3_, and the previously described LpxC inhibitors PF-4753299 (LpxC-2) and PF-5081090 (LpxC-4) [5]. Importantly, checkerboard assays were performed in RPMI 1640, as previously described [15], in order to perform assays under conditions that more closely mimic iron concentrations encountered by *A. baumannii* during infection. Minimal inhibitory concentrations for ATCC 19606 and ATCC 17978 for the compounds used in this study are shown in Table 1.

The fractional inhibitory concentration index (FICI) was calculated for each combination, and synergy was defined as a FICI ≤ 0.5, indifference as a FICI of >0.5 but ≤4 and antagonism as FICI > 4, as previously described [15]. As can be seen in Table 2, DFP did not demonstrate synergistic activity with either of the LpxC inhibitors. Synergistic activity with combinations of BIP and the LpxC inhibitors was strain-dependent, with LpxC-2 and BIP demonstrating synergy against the ATCC 19606 strain, and LpxC-4 and BIP demonstrating synergy against ATCC 17978. Interestingly, combinations of LpxC-2 or LpxC-4 with Ga(NO_3_)_3_ were highly synergistic for both reference strains (FICI values 0.08–0.38).

### 2.2. Time–Kill Analysis of Combinations of LpxC Inhibitors and Iron Chelators with A. baumannii Reference Strains

**Figure 1:**
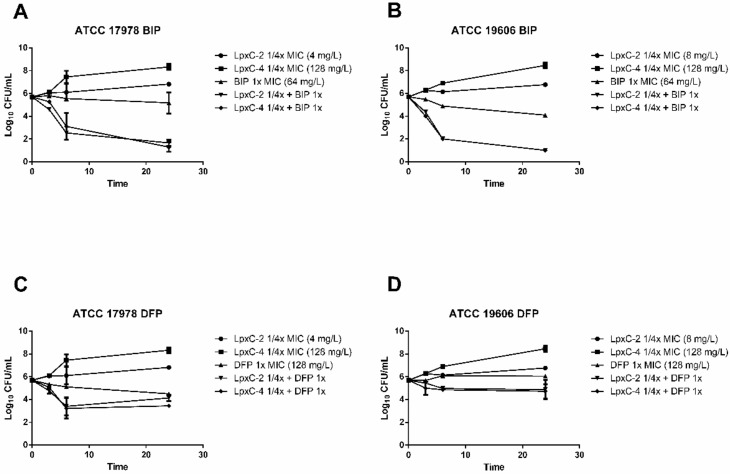
Time–kill curves for iron chelators in combination with LpxC inhibitors. Time–kill curves indicating bacterial counts in cultures at 0, 3, 6 and 24 h with the indicated *A. baumannii* strains containing combinations of BIP with LpxC-2 and LpxC-4 (**A**,**B**) or DFP with LpxC-2 and LpxC-4 (**C**,**D**). The indicated concentrations refer to the final concentration of each compound in the bacterial culture. BIP, 2,2′-bipyridyl; DFP, deferiprone. Time points indicate the mean bacterial count from a minimum of two biological replicates and error bars represent the standard deviation.

In order to further characterize the antimicrobial activity of combinations of LpxC inhibitors and iron chelators, time–kill studies were performed in RPMI medium. BIP and DFP were used at the MIC value and LpxC inhibitors were used at a concentration of 1/4 the MIC. Synergy was defined as a 2 log_10_ decrease in colony counts at 24 h with the combination of compounds compared to the most active single agent alone. As shown in Figure 1A,B, combinations of BIP with both LpxC inhibitors demonstrated synergistic activity, with a ~4 log_10_ reduction in bacterial counts compared to BIP alone in the case of the ATCC 17978 strain, and more than 3 log_10_ for the ATCC 19606 strain. The combinations of LpxC-2 and LpxC-4 with DFP reduced the growth of both strains with respect to the most active single compound, although this reduction was never greater than 1 log_10_ after 24 h of incubation (Figure 1C,D).

### 2.3. Time–Kill Analysis of Combinations of LpxC Inhibitors and Ga(NO_3_)_3_ with A. baumannii Reference Strains

**Figure 2:**
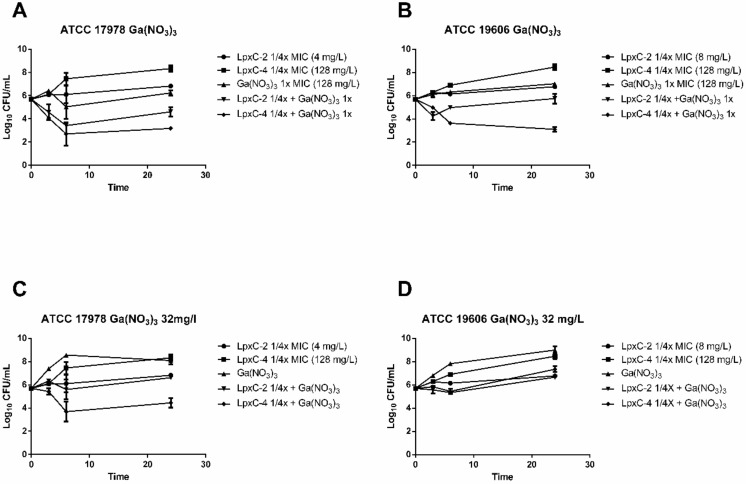
Time–kill curves for gallium nitrate in combination with LpxC inhibitors. Time–kill curves indicating bacterial counts in cultures at 0, 3, 6 and 24 h with the indicated *A. baumannii* strains containing combinations of Ga(NO_3_)_3_ with LpxC-2 and LpxC-4 (**A**–**D**) at the indicated concentrations. Time points indicate the mean bacterial count from a minimum of two biological replicates and error bars represent the standard deviation. Ga(NO_3_)_3_, gallium nitrate.

Time–kill studies were performed in RPMI medium with Ga(NO_3_)_3_ at the MIC value and LpxC inhibitors at a concentration of 1/4 the MIC, as described in the previous section. Combinations of Ga(NO_3_)_3_ with LpxC-2 and LpxC-4 decreased the growth of both strains compared to each compound separately; however, only the combination with LpxC-4 met the defined criteria for synergy (Figure 2A,B). In addition to the testing combinations of Ga(NO_3_)_3_ at the MIC (128 mg/L), synergistic activity of this compound with the LpxC inhibitors at 32 mg/L was determined since this concentration has previously been reported to be non-toxic in humans [16]. Ga(NO_3_)_3_ at this concentration, combined with LpxC-2 at 1/4x MIC, had no greater effect than the most active compound alone (Figure 2C,D), whereas this concentration in combination with LpxC-4 at 1/4x MIC reduced bacterial counts with respect to the most active single compound for both ATCC 19606 and ATCC 17978 strains (1.1 log_10_ and 3.6 log_10_, respectively). These results indicate that therapeutic concentrations of Ga(NO_3_)_3_ can act synergistically with subinhibitory concentrations of LpxC-4.

### 2.4. Activity of LpxC Inhibitors in Combination with Iron Chelators and Gallium Nitrate in A. baumannii Clinical Isolates

In order to determine if combinations of LpxC inhibitors and BIP or Ga(NO_3_)_3_ have synergistic activity against *A. baumannii* clinical isolates, checkerboard assays were carried out as described above with eight previously characterized clinical isolates [17,18]. DFP was not included in this analysis since it did not demonstrate synergy in either checkerboard or time–kill assays with the ATCC 17978 and ATCC 19606 reference strains. The activity of BIP in combination with LpxC-2 and LpxC-4 was strain-dependent, as the combination BIP/LpxC-2 was synergistic in 2 of 8 strains tested, and the BIP/LpxC-4 demonstrated synergy in 5 of 8 strains (Table 2). The synergistic activity of Ga(NO_3_)_3_ in combination with LpxC-2 was also strain-dependent, demonstrating synergy in 3 of 8 clinical isolates. Interestingly, Ga(NO_3_)_3_ in combination with LpxC-4 was highly synergistic against all clinical isolates, with FICI values between ≤0.09 and 0.31. These results indicate that some combinations of LpxC inhibitors with iron chelators or gallium demonstrate synergy in a strain-dependent manner, whereas the combination of LpxC-4 and Ga(NO_3_)_3_ was synergistic in all strains.

## 3. Discussion

The results of this study characterize novel synergistic combinations of LpxC inhibitors and compounds that interfere with bacterial iron metabolism, although the mechanisms underlying this synergistic activity are unclear. A previous study demonstrated that inhibition of LpxC in *A. baumannii* increases cell permeability and susceptibility to azithromycin, vancomycin and rifampin [19]. In this previous study, it was hypothesized that the increased cell permeability may contribute to the increased activity of these antimicrobials. Given that both BIP and Ga(NO_3_)_3_ have been proposed to act intracellularly [16,20], one possibility is that increased cell permeability due to LpxC inhibition permits greater intracellular accumulation of these compounds in *A. baumannii*. In contrast, the antimicrobial activity of deferiprone is thought to be primarily due to its iron-chelating activity in the extracellular space, which may explain why LpxC inhibition was less effective at increasing the activity of this compound.

LpxC inhibitors, iron chelators and gallium compounds are currently being developed as therapeutics for multidrug-resistant Gram-negative infections [2,10]. The results of this study indicate that combinations of these compounds may be synergistic against multidrug-resistant *A. baumannii* and may thus warrant further study. The combination of LpxC-4 together with gallium nitrate may be of particular interest given that it was highly synergistic against all strains tested. This study thus lays the groundwork for exploring synergistic combinations of LpxC inhibitors and compounds that interfere with iron metabolism, and may therefore have application in the clinical management of multidrug-resistant infections caused by *A. baumannii*.

## 4. Materials and Methods

### 4.1. A. baumannii Strains Used in This Study

The antibiotic-susceptible *A. baumannii* reference strains ATCC 19606 and ATCC 17978 were used. In addition, 8 previously characterized *A. baumannii* clinical isolates were employed in checkerboard assays [17,18]. All strains were maintained on LB agar and growth in LB broth before checkerboard and time–kill assays, unless otherwise stated.

### 4.2. LpxC Inhibitors, Iron Chelators and Gallium Nitrate

BIP, DFP and Ga(NO_3_)_3_ were purchased from Sigma and stock solutions were dissolved in water for DFP and Ga(NO_3_)_3_ and ethanol for BIP. The previously described LpxC inhibitors, PF-4753299 (LpxC-2) and PF-5081090 (LpxC-4) [5], were purchased from Axon Medchem (Groningen, Netherlands) and dissolved in DMSO. All reagents were prepared as 1024 mg/L stock solutions and stored at −20 °C until use.

### 4.3. Checkerboard Assays

Checkerboard assays were performed in RPMI 1640 as previously described [15], with modification. Briefly, bacterial cultures grown overnight on LB agar were re-suspended in RPMI, adjusted to an OD_600_ of 0.05–0.15 and diluted 1:10 in RPMI to yield a suspension of 1 × 10^6^ CFU/mL. Fifty µL of this suspension was combined with 50 µL of 7 two-fold dilutions of BIP, DFP or Ga(NO_3_)_3_ in combination with 9 two-fold dilutions of LpxC-2 or LpxC-4 and incubated at 37 °C for 24 h. The starting concentration for each compound was 256 mg/L. Growth inhibition was determined by visual inspection of turbidity in assay wells. All strains were assayed with at least two biological replicates.

### 4.4. Time–Kill Analysis

Time–kill assays were carried out in RPMI 1640 media with bacterial suspension at an initial concentration of 5 × 10^5^ CFU/mL in a final volume of 2 mL. BIP, DFP and Ga(NO_3_)_3_ were used at the MIC value and LpxC inhibitors were used at a concentration of 1/4 the MIC, unless otherwise indicated. Cultures were continuously shaken at 37 °C and samples were taken at 0, 3, 6 and 24 h for bacterial quantification on LB agar. Synergy was defined as a 2 log_10_ decrease in colony counts at 24 h with the combination of compounds compared to the most active single agent alone. All strains were assayed with at least two biological replicates.

## Figures and Tables

**Table 1:** MICs of iron chelators, gallium nitrate and LpxC inhibitors for a *A. baumannii* strains used in this study.

Strain	MIC (mg/L)				
	BIP	DFP	Ga(NO_3_)_3_	LpxC-2	LpxC-4
ATCC 17978	64	128	128	16	512
ATCC 19606	64	128	128	32	512
Ab-1	64	ND	128	16	>512
Ab-66	64	ND	128	32	>512
Ab-84	64	ND	128	16	>512
Ab-108	64	ND	32	16	>512
Ab-154	32	ND	64	16	512
Ab-167	64	ND	128	128	>512
Ab-176	64	ND	64	16	>512
Ab-208	64	ND	128	8	>512

BIP, 2,2′-bipyridyl; DFP, deferiprone; Ga(NO3)3, gallium nitrate; ND, not determined.

**Table 2:** Checkerboard assay for combinations of BIP, DFP and Ga(NO_3_)_3_ and LpxC inhibitors with a panel of *A. baumannii* strains.

Strain	BIP				DFP				Ga(NO_3_)_3_			
	LpxC-2		LpxC-4		LpxC-2		LpxC-4		LpxC-2		LpxC-4	
	FICI Value	Effect of Combination	FICI Value	Effect of Combination	FICI Value	Effect of Combination	FICI Value	Effect of Combination	FICI Value	Effect of Combination	FICI Value	Effect of Combination
ATCC 17978	0.53	Indifference	0.09	Synergy	1.01	Indifference	1.00	Indifference	0.38	Synergy	0.38	Synergy
ATCC 19606	0.50	Synergy	0.53	Indifference	1.00	Indifference	1.00	Indifference	0.27	Synergy	0.08	Synergy
Ab-1	1.02	Indifference	≤0.50	Synergy	ND	-	ND	-	1.00	Indifference	≤0.19	Synergy
Ab-66	0.38	Synergy	≤0.50	Synergy	ND	-	ND	-	0.38	Synergy	≤0.19	Synergy
Ab-84	1.00	Indifference	≤0.63	Indifference	ND	-	ND	-	0.50	Synergy	≤0.09	Synergy
Ab-108	0.63	Indifference	≤0.50	Synergy	ND	-	ND	-	0.75	Indifference	≤0.16	Synergy
Ab-154	0.56	Indifference	0.75	Indifference	ND	-	ND	-	0.53	Indifference	0.31	Synergy
Ab-167	0.50	Synergy	≤0.75	Indifference	ND	-	ND	-	0.53	Indifference	≤0.50	Synergy
Ab-176	0.52	Indifference	≤0.38	Synergy	ND	-	ND	-	0.50	Synergy	≤0.19	Synergy
Ab-208	0.53	Indifference	≤0.38	Synergy	ND	-	ND	-	0.75	Indifference	≤0.09	Synergy

BIP, 2,2′-bipyridyl; DFP, deferiprone; FICI, fractional inhibitory concentration index; Ga(NO_3_)_3_, gallium nitrate; ND, not determined.

## Data Availability

Data is available from the authors upon request.
